# Psychometric validation and measurement invariance of the Health-Promoting Lifestyle Profile II in Honduran university students

**DOI:** 10.1186/s12889-025-25899-9

**Published:** 2025-12-13

**Authors:** Marcio Alexander Castillo-Díaz, Carlos Alberto Henao Periañez

**Affiliations:** 1 Facultad de Ciencias Sociales, Departamento de Psicología/Maestría en Psicometría y Evaluación Educativa, Universidad Nacional Autónoma de Honduras, Tegucigalpa, Honduras; 2 Facultad de Salud, Escuela de Enfermería, Universidad del Valle, Cali, Valle del Cauca Colombia

**Keywords:** HPLP-II, Health behavior, Instrument validation, Psychometrics, University students

## Abstract

**Background:**

The Health-Promoting Lifestyle Profile II (HPLP-II) is an instrument that assesses health-promoting behaviors. Despite its extensive use in the international literature, studies in the Honduran context examining its psychometric properties and supporting its use as a culturally sensitive measure in lower-middle-income countries are lacking. This study aimed to evaluate (1) the structural validity and reliability of the HPLP-II in a sample of Honduran university students, (2) its measurement invariance across key sociodemographic variables, and (3) its convergent validity through associations with quality of life.

**Methods:**

This was a cross-sectional study conducted in a large national sample consisting of 6,012 Honduran university students. The participants completed a survey that included sociodemographic variables, the HPLP-II, and a measure of quality of life (WHOQOL-BREF). Structural and convergent validity were estimated via item-level confirmatory factor analysis models. Internal consistency was assessed through McDonald’s omega (Ω) and composite reliability (CR). Additionally, the configural, metric, and scalar invariance of the instrument were analyzed across different sociodemographic variables.

**Results:**

The six-factor correlated model of the HPLP-II demonstrated the best fit to the data (CFI = .968; TLI = .966; RMSEA = .066; SRMR = .061). Adequate factor loadings (λ > .30) and internal consistency indices (Ω > .700; CR > .800) were observed for each of the six factors of the instrument. The findings revealed configural, metric, and scalar invariance by sex, university campus, field of study, current occupation, and self-reported preexisting medical conditions. The results also revealed moderate to strong positive correlations between all dimensions of the HPLP-II and those of the WHOQOL-BREF (*r* > .300; *p* < .001).

**Conclusions:**

This study provides favorable evidence regarding the structural and convergent validity, as well as the reliability, of the scale for assessing health-promoting behaviors among Honduran university students. Measurement invariance up to the scalar level was supported, allowing for comparisons of latent means across subgroups. Finally, the evidence of convergent validity offers empirical support for the association between health-promoting behaviors and quality of life among university students.

**Supplementary Information:**

The online version contains supplementary material available at 10.1186/s12889-025-25899-9.

## Background

Noncommunicable chronic diseases (NCDs) have become a leading global health challenge. In 2021, in the Americas, NCDs were responsible for 6 million deaths, 38% of which occurred prematurely [1]. The increase in annual deaths attributable to NCDs is driven primarily by modifiable behavioral risk factors such as tobacco use, physical inactivity, unhealthy diets, and harmful alcohol consumption [2]. Reducing population exposure to these risk factors has been established as a global priority in the 2030 Agenda for Sustainable Development, which aims to reduce premature mortality from NCDs by one-third through the promotion of healthy lifestyles [3].

Health-promoting behaviors, as reflected in a healthy lifestyle, refer to the actions individuals take to enhance their emotional, physical, and spiritual well-being [4]. The evidence suggests that adopting a healthy lifestyle not only reduces the risk of NCDs but also enhances well-being and improves quality of life [5–8].

University students represent a particularly vulnerable group prone to adopting unhealthy lifestyles that increase the risk of developing NCDs [9, 10]. During this stage of the life cycle, students face multiple personal, academic, and social challenges that may negatively impact their physical and mental health, leading to the adoption of risk behaviors [11]. Recent studies have emphasized that sociodemographic, psychological, and contextual variables influence adherence to healthy behaviors among university students [12, 13], reinforcing the importance of assessing and promoting healthy habits within the university setting.

Investigating these behaviors requires instruments capable of providing reliable measurements, which can guide programs and policies. The most widely used reference instrument is the Health-Promoting Lifestyle Profile (HPLP) [14]. This instrument is based on Nola Pender’s health promotion model, which provides a conceptual framework for understanding how personal characteristics and cognitive-affective factors translate into health-promoting behaviors, with an emphasis on perceived benefits, self-efficacy, and interpersonal support [15]. The HPLP is a self-report questionnaire that quantifies the frequency of health-promoting behaviors. The original 48-item version identifies six dimensions derived from the health promotion model. It has preliminary evidence of content validity and adequate internal consistency, both in its original English version and in its initial Spanish adaptation [14, 16]. To enhance semantic precision and more fully incorporate the dimension of spirituality, the Health-Promoting Lifestyle Profile II (HPLP-II) was developed in 1996 [17]. This version consists of 52 items and is composed of six updated subscales: spiritual growth, health responsibility, physical activity, nutrition, interpersonal relationships, and stress management. Factor analyses confirmed its six-factor correlated structure and satisfactory internal consistency [17].

The HPLP-II has been validated in various international contexts. In Europe, studies have been conducted in Portugal [18], Spain [19] and Italy [20]; in Asia, studies have been conducted in countries such as Iran [21], Malaysia [22, 23], Taiwan [24], China [25], South Korea [26], and Sri Lanka [27]; and in Africa, there is evidence of validation in South Africa [28]. In Latin America, Spanish-language adaptations have been developed in countries such as Mexico [29] and Colombia [30, 31]. In addition, recent evidence of validity has also been reported in the United States [32]. In applied contexts, the HPLP-II has been used in clinical settings with participants presenting specific comorbidities, such as hypertension [33, 34], diabetes [35], and cancer [36, 37]. It has also been applied across diverse population groups, including older adults [27, 34], pregnant women [38], and young individuals, such as university students [12, 39].

Although evidence on the validity of the HPLP-II among Latin American university populations remains emerging, some studies have supported the use of this instrument. Recently, the validity of the Spanish version of the HPLP-II was evaluated in Colombian university students, confirming a six-factor structure with adequate model fit indices and high internal consistency [31]. In contrast, a subsequent study with a different sample of Colombian students identified a five-factor solution after merging and redistributing certain items, suggesting potential cultural or contextual particularities [30]. In Mexico, similar psychometric properties were confirmed for the Spanish version of the instrument, which also supports the six-factor model [29].

To the best of our knowledge, studies of the HPLP-II in Latin American university samples have focused primarily on the factorial structure of the scale, with a scarcity of evidence regarding its measurement invariance across different population groups and its relationship with other relevant variables, such as quality of life. Studying measurement invariance based on sociodemographic characteristics is essential, as evidence indicates differences in the adoption of health-promoting behaviors between urban and rural contexts [37], as well as by gender, with lower scores observed particularly among women [40, 41]. However, to make valid comparisons of latent HPLP-II scores across these and other sociodemographic variables, it is necessary to examine the measurement invariance of the instrument across different population groups.

Given this context, the objectives of this study are (1) to analyze the structural validity and reliability of the HPLP-II in a large sample of Honduran university students; (2) to assess the measurement invariance of the instrument across sociodemographic variables (i.e., age, sex, university campus, field of study, current occupation, and self-reported preexisting medical conditions); and (3) to provide evidence of the scale’s convergent validity through its association with quality of life. Based on the current evidence, this is the first study to evaluate the psychometric properties of the HPLP-II in the Honduran context. By providing evidence of the structural and convergent validity, reliability, invariance, and cultural appropriateness of the HPLP-II in a university population, this study offers an empirical foundation for the implementation of the scale in this context, enabling the monitoring of health-promoting lifestyles. Furthermore, the use of scale scores may support the design of evidence-based interventions in university settings.

## Methods

### Study design and participants

This cross-sectional study followed the methodological recommendations proposed by the COSMIN initiative (Consensus-based Standards for the Selection of Health Measurement Instruments) for the evaluation of the psychometric properties of health-related instruments [42]. The study population consisted of university students from a major public university in Honduras, the country’s leading higher education institution, which accounts for 35% of the national total number of higher education students [43]. The total first-year student enrollment at the institution was 13,379 in 2024 and 9,471 during the first academic term of 2025 [44]. The study sample was selected via nonprobability convenience sampling. The inclusion criteria were active enrollment at any of the university campuses nationwide (one main campus and eight regional centers across the country), current first-year status in an undergraduate program, enrollment in any academic discipline, and being 18 years of age or older.

The data for this study derive from a large-scale project titled “Psychosocial Determinants of Well-being and Quality of Life in University Students: A Diagnostic and Longitudinal Evaluation”, which was approved by the Research Ethics Committee of the Faculty of Social Sciences at the National Autonomous University of Honduras (Ref. CEIFCS-2024-P01). The study was conducted in strict accordance with the Declaration of Helsinki and its subsequent amendments. All the participants provided digital informed consent, and their participation was contingent upon their acceptance. The consent form outlined the purpose of the study, the voluntary nature of participation, the right to withdraw at any time, and the guarantee of data privacy and anonymity.

Data collection was carried out virtually through an electronic form distributed via official institutional communication channels, which targeted first-year university students. The form was disseminated nationally across all university campuses, which span the entire country. The general survey included sociodemographic questions and a series of psychometric instruments. The dataset analyzed in this study corresponds to students who completed the form between March 2024 and February 2025. Details on the final sample size are provided in the Results section.

### Measures

Sociodemographic information related to participant characteristics was collected via a screening instrument. For this study, the following variables were considered: age, sex, university campus, field of study, current occupation, and self-reported preexisting medical conditions.

Health-Promoting Lifestyle Profile II (HPLP-II). This instrument was developed by Walker et al. [14, 17]. This study uses the Spanish version of the scale, which has been validated in Latin American higher education contexts [29, 31]. The scale consists of 52 items rated on a four-point Likert scale ranging from “never” to “routinely.” The theoretical model underlying the scale groups the items into six dimensions: physical activity (8 items), stress management (8 items), interpersonal relationships (9 items), spiritual growth (9 items), nutrition (9 items), and health responsibility (9 items). Higher scores reflect greater engagement in health-promoting behaviors within each subscale. The use of the HPLP-II in this study complies with the research purposes authorized by its original developers, who retain the copyright of all versions.

World Health Organization Quality of Life-BREF (WHOQOL-BREF). This scale was developed by the World Health Organization (WHO) and consists of 26 items rated on a five-point Likert scale [45, 46]. It includes two general items on overall quality of life and satisfaction with health status and 24 items grouped into four specific domains: physical (7 items), psychological (6 items), social (3 items), and environmental (8 items). Higher scores in each dimension indicate a greater presence of the corresponding latent construct. Evidence from meta-analyses supports the structural validity and reliability of the four-dimensional model [47].

We used Spanish versions of the HPLP-II [29] and WHOQOL-BREF [48]. Prior to administration, both instruments were qualitatively reviewed by three expert judges—a psychologist, a nurse, and a physician. The review specifically analyzed semantic (accuracy of meaning), idiomatic (colloquial language use), experiential (relevance to the students’ daily lives), and conceptual (consistency of the underlying constructs) equivalence of each item. This procedure ensured that the phrasing was both linguistically precise and culturally significant for Honduran university students. In addition, a pilot test was conducted with 15 students from the target population to assess item clarity and comprehension. No modifications were deemed necessary following these evaluations, confirming the adequacy of the Spanish versions for the Honduran context.

### Data analysis

Data analyses were conducted via R software (version 4.4.2) [49] within the RStudio environment (version 2025.5.1.513) [50]. Various analytical packages have been employed, including tidyverse [51], semTools (version 0.5–6) [52], and lavaan (version 0.6–16) [53]. The dataset was examined prior to analysis, and no missing values were identified. Descriptive statistics for sociodemographic characteristics are presented as frequencies and percentages for categorical variables, and as means, standard deviations, minimum, and maximum values for continuous variables. Following recommendations for reporting data derived from Likert-type scales, a preliminary descriptive analysis was conducted to examine the distribution of responses across all categories for each item [54, 55]. This procedure ensures the preservation of information integrity at each response point on the scale. The literature suggests that each response category should ideally have a minimum frequency of 10%, as lower frequencies may negatively affect model estimation [56].

The psychometric properties of the instrument were evaluated in several phases. First, the dimensionality of the HPLP-II was assessed through confirmatory factor analysis (CFA). In this phase, the original structure of the scale, which comprises six correlated latent variables accounting for the variance of the observed indicators (items), was tested [14]. To examine the empirical plausibility of a general factor underlying health-promoting behaviors, we also tested a unidimensional, hierarchical, and bifactor model. The unidimensional model posits that a single latent variable accounts for the variance of all 52 items on the scale. In contrast, the hierarchical model specifies six first-order latent variables explained by a single second-order general factor. On the other hand, the bifactor model specifies that each item loads simultaneously on a general factor, representing the common variance across all health-promoting behaviors, and on one of the six orthogonal specific factors, which capture the unique variance associated with each dimension.

The weighted least squares mean- and variance-adjusted estimator (WLSMV) was employed as the estimation method for all models. According to the literature, this estimator is considered the most appropriate for parameter estimation in models comprising ordinal polytomous items [57]. Model fit was evaluated via the comparative fit index (CFI), Tucker–Lewis’s index (TLI), the root mean square error of approximation (RMSEA), and the standardized root mean square residual (SRMR). Acceptable model fit was indicated by CFI and TLI values ≥ 0.95, and RMSEA and SRMR values ≤ 0.08 [58]. Model selection was guided by comparing these fit indices, favoring the model with higher CFI and TLI values and lower RMSEA and SRMR values [59].

Factor loadings of λ ≥ 0.40 (*p* < 0.05) were considered acceptable indicators of measurement model adequacy. The internal consistency of the identified latent dimensions was assessed via McDonald's omega (Ω) and composite reliability (CR). Values of both coefficients ≥ 0.70 were interpreted as acceptable indicators of reliability [60, 61]. Additionally, we reported the average variance extracted (AVE), which contemporary literature has suggested functions as an indicator of precision [62]. Values above 0.50 are generally considered acceptable cutoff points [63].

Second, a multigroup confirmatory factor analysis (MG-CFA) was conducted to assess measurement invariance based on sex (male and female), regional campus (main campus, primary regional campus, and other regional campuses grouped), area of study (four academic areas offered by the university), current occupation (studying only vs. studying and working), and self-reported preexisting medical conditions (yes or no). Three nested models were tested by progressively imposing parameter constraints to evaluate different levels of invariance: (1) configural invariance, which assesses whether the factor structure (i.e., dimensions) is equivalent across groups; (2) metric invariance, which adds the constraint of equal factor loadings across groups; and (3) scalar invariance, which assumes equal item intercepts in addition to the constraints of the previous levels [64]. Configural invariance was evaluated using the same criteria as the initial CFA. Metric and scalar invariance were assessed sequentially by comparing each model with its immediately preceding model. Evidence of invariance was determined based on changes in fit indices: ΔCFI ≤ 0.010 and ΔRMSEA ≤ 0.015 [64, 65].

Finally, to provide evidence of the validity of the HPLP-II in relation to an external variable, we employed a structural equation modeling (SEM) approach. First, we estimated a four-factor correlated measurement model of the WHOQOL-BREF. Subsequently, we conducted a SEM model that tested the correlations between the latent dimensions measured by the HPLP-II and the latent dimensions of quality of life assessed by the WHOQOL-BREF. The selection of the WHOQOL-BREF as an indicator of convergent validity was based on prior literature supporting the empirical association between these constructs in university students [5, 8, 66–68]. Unlike simple correlations based on observed scores, correlations derived from latent variable models account for item factor loadings and measurement error, thereby providing more accurate information about the relationships between latent constructs [69]. Model fit was evaluated using the same criteria applied in the initial CFA. Statistically significant correlations between latent variables were considered at the *p* < 0.05 level. Correlation coefficients of approximately 0.10, 0.30, and 0.50 were interpreted as small, moderate, and strong, respectively [70].

## Results

### Sample characteristics

The study sample consisted of 6,012 university students, representing 26% of the first-year student cohorts from 2024 to 2025. Table 1 presents the sociodemographic characteristics of the sample. The sample consisted predominantly of young adults, with women representing 65.40% of the participants. More than three-fifths of the respondents were enrolled at the university’s main campus. Specifically, the central campus (Tegucigalpa) had a target population of 13,514 students, of whom 3,890 participated (28.8%). The main regional campus (San Pedro Sula) had 4,710 students, with 1,077 respondents (22.9%), while the medium and small regional campuses had a combined population of 4,626 students, with 1,045 respondents (22.6%). Although this distribution indicates an overrepresentation of the central campus in the analytic sample, it is consistent with the fact that this campus also concentrates the largest share of the university’s total first-year student population. Regarding field of study, the sample comprised students from all academic areas offered by the university, with the majority enrolled in economic and administrative sciences (42.28%). One-fifth of the participants reported combining work and study, and approximately one in ten self-reported at least one preexisting medical condition.

**Table 1:** Sociodemographic characteristics of the sample (n = 6,012)

Variables	(M ± SD)/n (%)
*Age*	21.59 ± 3 (range = 18–48)
*Sex*	
Women	3,932 (65.40%)
Men	2,022 (33.63%)
Nonbinary	58 (0.97%)
*University campus*	
Central Campus (Tegucigalpa)	3,890 (64.71%)
Main regional campus (San Pedro Sula)	1,077 (17.91%)
Medium and small regional campuses	1,045 (17.38%)
*Field of study*	
Social Sciences, Humanities, and Arts	1,217 (20.25%)
Engineering and Physical–Mathematical Sciences	1,089 (18.11%)
Economics and business sciences	2,542 (42.28%)
Biological and health sciences	1,164 (19.36%)
*Current occupation*	
Just study	4,789 (79.66%)
Study and work	1,223 (20.34%)
*Self-reported preexisting medical conditions *	
Yes	772 (12.84%)
No	5,240 (87.16%)

*M* Mean, *SD* Standard deviation

### Preliminary analysis

**Fig. 1:**
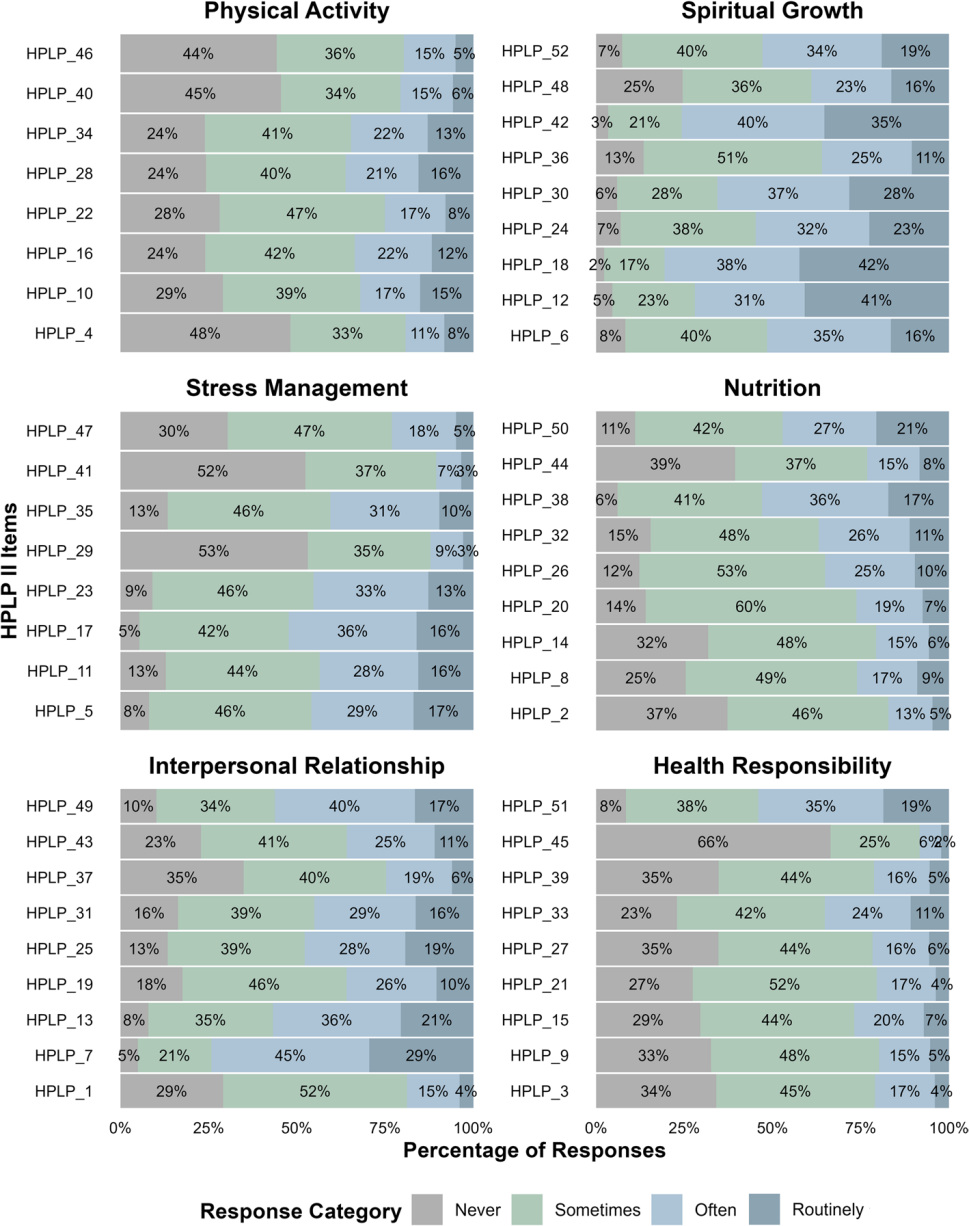
Response percentages by likert scale category for each item of the HPLP-II

Figure 1 displays the response percentages for each category of the HPLP-II items. Overall, variability was observed in the response patterns across items within the six dimensions. None of the items showed a complete absence of endorsement for any response category. However, some items had response rates below 10% in the extreme categories, with “Routinely” being the least endorsed category across all dimensions, except for the items in the spiritual growth dimension. The items in the physical activity and health responsibility dimensions revealed a pattern of endorsement concentrated in the low- to moderate-frequency categories (“Never” and “Sometimes”). In contrast, items theoretically representing the stress management, interpersonal relationship, and nutrition dimensions tended to be endorsed in the intermediate categories (“Sometimes” and “Often”). Finally, items from the spiritual growth dimension exhibited a response trend favoring higher-frequency categories (“Often” and “Routinely”).

### Confirmatory factor analysis

The fit indices for the different factorial structures of the HPLP-II are presented in Table 2. The results indicate an unacceptable model fit for the unidimensional structure (CFI & TLI < 0.95; RMSEA & SRMR > 0.08). In contrast, both the six-correlated factors model and the hierarchical model demonstrated acceptable fit (CFI & TLI > 0.95; RMSEA & SRMR < 0.08). However, the correlated six-factor model showed higher CFI and TLI values (0.968 & 0.966) and lower RMSEA and SRMR values (0.066 & 0.061) and was therefore retained for subsequent analyses. Although a bifactor model was also tested, it did not converge to a proper solution (non-positive definite covariance matrix), and therefore no fit indices are reported.

**Table 2:** Fit indices from the confirmatory factor analysis of the HPLP-II

Model	χ^2^ (df)	CFI	TLI	RMSEA (90% CI)	SRMR
Unidimensional	73,500.144 (1274)*	.891	.908	.097 (.098 –.100)	.085
Correlated factors	34,640.167 (1259)*	.968	.966	.066 (.066 –.067)	.061
Hierarchical	40,892.006 (1268)*	.962	.960	.072 (.072 –.073)	.067
Bifactor	NA	NA	NA	NA	NA

NA Fit indices not available because the bifactor model did not converge (non-positive definite covariance matrix). Recommended cutoffs: CFI ≥.95, TLI ≥.95, RMSEA ≤.08, SRMR ≤.08

* = *p* value <.001

**Fig. 2:**
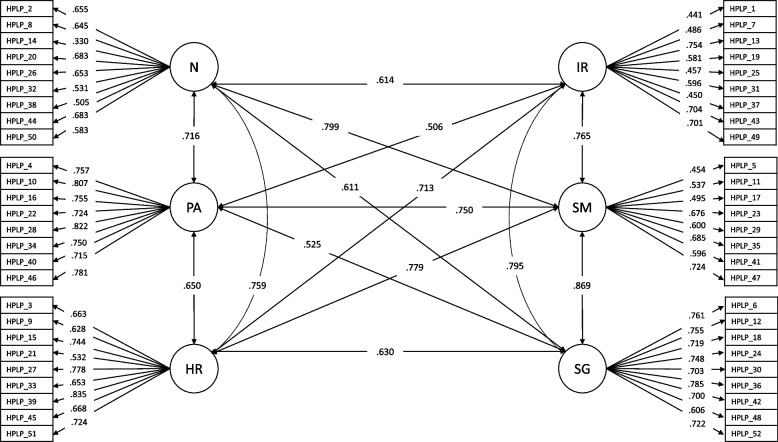
Standardized factor loadings and interfactor correlations from the six-correlated factor model

Figure 2 displays the CFA model with the best fit (correlated factors model), including standardized factor loadings and interfactor correlations. The results revealed statistically significant and adequate loadings (*p* < 0.001), ranging from 0.330 to 0.683 for nutrition (mean λ = 0.585), from 0.715 to 0.822 for physical activity (mean λ = 0.764), from 0.532 to 0.835 for health responsibility (mean λ = 0.692), from 0.441 to 0.754 for interpersonal relationships (mean λ = 0.572), from 0.454 to 0.724 for stress management (mean λ = 0.596), and from 0.606 to 0.785 for spiritual growth (mean λ = 0.722).

Interfactor correlations ranged from 0.506 to 0.869, all of which were statistically significant (*p* < 0.001), indicating strong but distinct associations among the six dimensions. All the subscales also demonstrated acceptable reliability coefficients: nutrition (Ω = 0.788; CR = 0.827), physical activity (Ω = 0.892; CR = 0.918), health responsibility (Ω = 0.863; CR = 0.893), interpersonal relationships (Ω = 0.786; CR = 0.817), stress management (Ω = 0.772; CR = 0.817), and spiritual growth (Ω = 0.878; CR = 0.908). The AVE showed values above 0.50 for physical activity (0.585) and spiritual growth (0.524), and values below the acceptable cutoff for stress management (0.363), nutrition (0.354), interpersonal relationships (0.341), and health responsibility (0.486).

### Measurement invariance

Table 3 presents the results of the multigroup confirmatory factor analysis (MG-CFA) used to assess measurement invariance in the study sample. The findings support configural, metric, and scalar invariance (ΔCFI < 0.010; ΔRMSEA < 0.015) of the HPLP-II across all tested variables: sex, university campus, field of study, current occupation, and self-reported preexisting medical conditions.

**Table 3:** Measurement invariance of the HPLP-II in Honduran university students

Invariance Model	χ^2^(df)	Δχ^2^(Δdf)	CFI	ΔCFI	RMSEA (90% CI)	ΔRMSEA
Invariance across sex						
Configural	36,181.436 (2518)	-	.968	-	.067 (.066 –.068)	-
Metric	37,111.635 (2564)	930.199 (46)	.967	**.001**	.067 (.067 –.068)	**.000**
Scalar	37,688.775 (2662)	577.14 (144)	.966	**.001**	.066 (.066 –.067)	**.001**
Invariance across university campus						
Configural	37,791.481 (3777)	-	.968	-	.067 (.066 –.068)	-
Metric	38,753.464 (3869)	961.983 (92)	.967	**.001**	.067 (.066 –.068)	**.000**
Scalar	38,465.122 (4065)	288.342 (196)	.968	**.001**	.065 (.064 –.066)	**.002**
Invariance across field of study						
Configural	39,533.207 (5036)	-	.967	-	.068 (.067 –.068)	-
Metric	41,950.362 (5174)	2417.155 (138)	.965	**.002**	.069 (.068 –.069)	**.001**
Scalar	41,187.405 (5468)	702.957 (294)	.966	**.001**	.066 (.065 –.067)	**.003**
Invariance across current occupation						
Configural	36,482.636 (2518)	-	.968	-	.067 (.066 –.068)	-
Metric	37,940.982 (2564)	1458.346 (46)	.966	**.002**	.068 (.067 –.068)	**.001**
Scalar	37,333.472 (2662)	607.510 (98)	.967	**.001**	.066 (.065 –.066)	**.002**
Invariance across self-reported preexisting medical conditions						
Configural	36,048.341 (2518)	-	.968	-	.067 (.066 –.067)	-
Metric	36,640.165 (2564)	591.824 (46)	.968	**.000**	.067 (.066 –.067)	**.000**
Scalar	36,530.642 (2662)	109.523 (98)	.968	**.000**	.065 (.064 –.066)	**.002**

Bolded values indicate support for measurement invariance according to recommended cut-off criteria (ΔCFI ≤.010; ΔRMSEA ≤.015)

### Evidence of validity in relation to quality of life

A CFA of the WHOQOL-BREF was conducted to evaluate its measurement model. The four-factor structure showed good fit to the data (χ^2^ = 11,764.763; df = 246; *p* < 0.001; CFI = 0.970; RMSEA = 0.077 [90% CI = 0.076–0.080]; SRMR = 0.066), with factor loadings λ > 0.40 and acceptable reliability indices by domain: physical (Ω = 0.719), psychological (Ω = 0.843), social (Ω = 0.697), and environmental (Ω = 0.823).

The structural equation model examining the correlations between the HPLP-II and the WHOQOL-BREF also demonstrated good fit to the data (χ^2^ = 67,198.197; df = 2,729; *p* < 0.001; CFI = 0.966; TLI = 0.964; RMSEA = 0.063 [90% CI = 0.062–0.063]; SRMR = 0.058). Table 4 revealed moderate to strong positive correlations between all HPLP-II dimensions and those of the WHOQOL-BREF (*r* > 0.300; *p* < 0.001). The strongest correlations between dimensions of the two instruments were observed between spiritual growth and the psychological domain (*r* = 0.840, *p* < 0.001), and the physical domain (*r* = 0.696, *p* < 0.001); between interpersonal relationships and the social domain (*r* = 0.717, *p* < 0.001); and between stress management and both the physical (*r* = 0.664, *p* < 0.001) and the psychological domain (*r* = 0.653, *p* < 0.001).

**Table 4:** Correlations between the latent dimensions of the HPLP-II and the WHOQOL-BREF

Factor	PA	N	HR	SM	IR	SG	PD	SD	PsD	ED
HPLP-II										
PA	-									
N	.714*	-								
HR	.649*	.757*	-							
SM	.747*	.799*	.777*	-						
IR	.505*	.765*	.712*	.615*	-					
SG	.525*	.613*	.630*	.871*	.795*	-				
WHOQOL-BREF										
PD	.384*	.440*	.391*	.664*	.499*	.696*	-			
SD	.303*	.367*	.428*	.523*	.717*	.609*	.694*	-		
PsD	.347*	.403*	.432*	.653*	.549*	.840*	.855*	.770*	-	
ED	.323*	.455*	.408*	.570*	.530*	.613*	.770*	.771*	.691*	-

*PA* Physical activity, *N* Nutrition, *HR* Health responsibility, *SM* Stress management, *IR* Interpersonal relationships, *SG* Spiritual growth, *PD* Physical domain, *SD* Social domain, *PsD* Psychological domain, *ED* Environmental domain

* = *p* value <.001

## Discussion

The results of this study provide evidence of the psychometric properties of the HPLP-II in a sample of 6,012 Honduran university students. The findings support the structural validity of the scale, the reliability of scores across all dimensions, its measurement invariance across key sociodemographic characteristics, and its convergent validity through associations with the WHOQOL-BREF.

The descriptive analysis of item response categories indicated a tendency toward higher frequencies in the lower and intermediate response options. This pattern aligns with previous findings suggesting that university students represent a population at risk for low adherence to health-promoting behaviors [10, 12]. However, the items from the spiritual growth dimension tended toward greater endorsement in the medium and high response categories. This finding may reflect a sociocultural nuance characteristic of the Latin American context, where spirituality is a deeply internalized and socially reinforced construct [71]. At the same time, some response categories—specifically the extreme options—did not reach the 10% frequency threshold that we initially considered desirable for ordinal indicators. This distributional pattern is consistent with what would be expected in self-report assessments of health behaviors, where university respondents often avoid extreme categories and gravitate toward lower-to-middle options [12]. From a modeling perspective, the use of the WLSMV estimator and the large sample size likely mitigated the impact of sparse cells on parameter estimation; however, future applications should continue to monitor category functioning and consider collapsing rarely endorsed categories when empirically and conceptually justified.

Regarding the validity results based on the internal structure of the scale, both the six-factor correlated model, and the hierarchical model demonstrated acceptable fit indices (TLI & CFI > 0.95; RMSEA & SRMR < 0.08). Although neither model was rejected, the correlated factor model showed improved global fit, with a ΔCFI = 0.006 and a reduction in RMSEA of Δ = 0.006. Research based on simulation studies indicates that, in large samples (n > 1,000), even a CFI difference of 0.005 can represent a meaningful improvement in model fit [61]. In addition, the six-factor correlated model demonstrates a stronger theoretical alignment with the original structure proposed for the scale [17] and is consistent with recent research that has tested the factorial structure of the HPLP-II in university student populations, yielding comparable fit indices and factor reliability estimates [29, 31].

The interpretation of fit indices should be considered in light of model complexity and sample size. Our models included 52 items and a very large sample (n = 6,012), conditions under which conventional cutoffs (e.g., CFI ≥ 0.95, RMSEA ≤ 0.08) may yield overly stringent or misleading conclusions [72]. For this reason, we also reported the SRMR, which has been described as relatively less sensitive to sample size and model complexity [73]. The SRMR values obtained were within the recommended thresholds (≤ 0.08), supporting the adequacy of model fit in addition to the other indices.

The six correlated-factors model revealed interfactor correlations ranging from 0.506 to 0.869. This range suggests that each dimension retains a specific variance, which may serve as a differentiated target for designing more focused and operationally feasible interventions. For example, a randomized controlled trial involving patients with metabolic syndrome demonstrated that a program based on the health promotion model significantly increased scores across each of the six HPLP-II subscales over a two-year follow-up [74]. These findings support the feasibility and clinical effectiveness of interventions tailored to specific domains of health-promoting behaviors.

With respect to factor loadings, almost all items showed standardized loadings above the 0.40 criterion specified a priori, with only one item falling slightly below this threshold (HPLP_14: “daily consumption of cereals and grains”), which yielded a loading of λ = 0.330. We decided to retain this item because it is conceptually central to its domain, its removal did not improve overall model fit or the reliability of the corresponding subscale, and similar patterns have been documented in previous validations of the HPLP-II [31].

In terms of reliability, all factors demonstrated adequate Ω and CR reliability coefficients (both > 0.70), indicating satisfactory internal consistency. These values are consistent with those reported in Malaysian and Colombian university student samples [22, 31]. However, several domains—particularly stress management, nutrition, interpersonal relationships, and health responsibility—showed AVE values below the 0.50 threshold. Although both AVE and CR are calculated using factor loadings and measurement error (or residual variance), differences between these indices help explain the discrepancy observed in internal consistency estimates. AVE is a more stringent indicator that focuses on the variance explained by the latent construct and is highly sensitive to the magnitude and heterogeneity of the factor loadings [62]. CR, on the other hand, is a broader measure of internal consistency, more strongly influenced by the number of items and less affected by loading heterogeneity [63]. While CR and Ω support the adequacy of the six HPLP-II dimensions, the lower AVE values suggest that some domains may encompass more diverse behavioral expressions. Future research should examine whether these patterns replicate across samples and evaluate whether item refinement or alternative modeling strategies could strengthen precision.

Although a bifactor model (general + six specific factors) was tested, it did not converge to a proper solution, suggesting that the specification of a general factor orthogonal to the six domains was not empirically viable in our data. This finding may be due to the high interfactor correlations and the restricted unique variance of some domains. Future research could benefit from testing more flexible approaches, such as bifactor-ESEM models, which allow cross-loadings and can provide a more accurate representation of the interplay between a general factor and domain-specific dimensions.

The results of the multigroup confirmatory factor analysis supported measurement invariance of the HPLP-II across sex, university campus, academic discipline, current occupational status, and self-reported preexisting medical conditions. This finding indicates that the six-factor structure, item loadings, and intercepts of the scale operate equivalently across all contrasted subgroups. While previous studies have explored sociodemographic differences in health-promoting behaviors [12, 40], to the best of our knowledge, this is the first study to provide empirical evidence of measurement invariance of the HPLP-II in a sample of Latin American university students. As emphasized in the literature, such evidence supports the validity of unbiased latent comparisons between groups, thereby enabling the detection of true differences in the latent dimensions assessed by the scale [64].

The positive correlation ranging from moderate to strong (*r* > 0.300) between all the HPLP-II subscales and the WHOQOL-BREF domains provides evidence of the scale’s convergent validity. As postulated by Pender’s health promotion model, health-promoting behaviors translate into greater perceived well-being and quality of life [15]. Similar findings have been reported in studies assessing the association between health-promoting behaviors and quality of life in university students from different countries [66–68].

The strongest correlations were observed between spiritual growth and the psychological (*r* = 0.840) and physical (*r* = 0.696) domains of the WHOQOL-BREF. This finding aligns with research conducted in Asian populations, where the spiritual dimension emerged as a strong predictor of psychological well-being [27]. Spiritual growth is considered a key component of a health-promoting lifestyle and has been associated with the reduction of harmful behaviors and the improvement of well-being [75, 76]. On the other hand, the strong association between interpersonal relationships and the social domain (*r* = 0.717) confirms that perceived social support is a key component of social well-being during the university stage [28]. Finally, the correlations between stress management and the physical (*r* = 0.664) and psychological (*r* = 0.653) domains suggest that the ability to manage stress impacts not only mental health but also perceptions of physical well-being—an observation that is consistent with prior literature [27].

### Implications and limitations of the study

These findings reinforce the utility of the HPLP-II as a valid tool for monitoring the adoption of health-promoting behaviors, enabling the identification of deficient domains and at-risk subgroups within university settings. The evidence of convergent validity with quality of life may inform the design of interventions aimed at enhancing health-promoting behaviors and their impact on overall perceived well-being. Furthermore, the results provide a comparative framework for future research in Latin America, contributing to the development of coordinated regional health promotion policies.

Several limitations and avenues for future research should be noted. First, although the large sample size increases the statistical power of the findings, the use of nonprobability sampling limits the generalizability of the results. Despite the proportional representation of campuses in the sample, the central campus was overrepresented, and selection bias may have occurred due to factors such as internet access and self-selection (volunteering). Therefore, caution is warranted when generalizing these findings. Future research may consider the use of post-stratification weights to better align the sample with the underlying population, as well as the implementation of probabilistic sampling strategies. Moreover, future studies should assess the scale's psychometric properties in other higher education settings (e.g., different institutions, historically underserved groups) and in populations beyond the university context, such as clinical samples or individuals with varying levels of educational attainment.

Second, the cross-sectional design of this study precludes causal inferences regarding the relationship between health-promoting behaviors and quality of life and does not capture intraindividual variation in HPLP-II scores throughout the university experience. Longitudinal research is recommended to assess the instrument’s test–retest reliability, map the relationship between HPLP-II scores and relevant outcomes (e.g., well-being, mental health), and examine measurement invariance across different stages of the academic trajectory.

Third, although measurement invariance testing supported configural, metric, and scalar invariance, strict invariance (equal residual variances) was not tested. While scalar invariance allows comparisons of latent means, full comparability of observed scores would require evidence of strict invariance [77]; therefore, future research should explore this level of invariance.

Fourth, this study focused on the original 52-item version of the scale. Although our findings support the adequate psychometric properties of the six correlated factors model, future research could explore the psychometric adaptation of abbreviated versions of the instrument [20, 25] in Latin American contexts. The use of shorter versions may facilitate data collection involving multiple instruments and help reduce respondent fatigue commonly associated with lengthy self-report questionnaires.

Fifth, the exclusive reliance on self-report instruments may have introduced social desirability bias, and the absence of response-time checks could also have affected response quality, potentially inflating scores or reflecting inattentive responding. Future applications should consider including objective health indicators (e.g., body mass index and blood pressure) and incorporating response-time controls to strengthen criterion validity and data quality. In addition, because both the HPLP-II and the WHOQOL-BREF were self-report measures administered in the same session, common method bias may have inflated associations between constructs, and this should be considered when interpreting the findings.

Finally, we recommend the use of alternative psychometric approaches, such as item response theory (IRT) and network analysis, to evaluate the HPLP-II from different perspectives. These methods could enrich the psychometric evidence at the item level by identifying parameters of discrimination and difficulty or by detecting items with high centrality and key interconnections. Furthermore, such approaches may be particularly useful for clarifying the sources of variability underlying the lower AVE values observed in some domains, as IRT can disentangle item-level precision and network models can reveal structural heterogeneity across behaviors These insights could optimize the measurement process, especially for designing targeted preventive and clinical interventions tailored to individual needs.

## Conclusion

This study provides the first psychometric evidence of the HPLP-II in a large sample within the Honduran context. The findings support the structural and convergent validity, as well as the reliability of the scale for assessing health-promoting behaviors among university students in Honduras. Measurement invariance across different sociodemographic variables supports latent means comparisons of scale scores across population subgroups. Furthermore, evidence of convergent validity offers empirical endorsement for the association between health-promoting behaviors and quality of life in this population. The implementation of the HPLP-II can inform the diagnosis and evaluation of evidence-based intervention programs aimed at promoting health-enhancing behaviors among university students.

## Supplementary Information

Supplementary Material 1.

## Data Availability

The datasets used and analyzed during the current study are available from the corresponding author upon reasonable request.

## Abbreviations

AVE: Average variance extracted
CFA: Confirmatory factor analysis
CFI: Comparative fit index
CR: Composite reliability
COSMIN: Consensus-based standards for the selection of health measurement instruments
HPLP-II: Health-Promoting Lifestyle Profile II
MGCFA: Multigroup confirmatory factor analysis
RMSEA: Root mean square error of approximation
SEM: Structural equation model
TLI: Tucker–lewis index
WHOQOL-BREF: World Health Organization Quality of Life instrument BREF version
