# Supplementary material for: Psychometric validation and measurement invariance of the Health-Promoting Lifestyle Profile II in Honduran university students
Source: BMC Public Health. 2025 Dec 13;26:229. doi: 10.1186/s12889-025-25899-9 (PMC12817687; doi:10.1186/s12889-025-25899-9)
Supplement: Supplementary file 1 — Supplementary Material 1. [file 12889_2025_25899_MOESM1_ESM.docx]

**Supplementary Data 1- Syntax for psychometric analyses**

Psychometric validation and measurement invariance of the Health-Promoting Lifestyle Profile II in Honduran university students

**Supplementary Data 1- Syntax for psychometric analyses**

This supplementary file contains the R syntax used to conduct the analyses reported in the article *“Psychometric validation and measurement invariance of the HPLP-II in Honduran university students.”* It includes code for structural validity, reliability estimation, multigroup measurement invariance across sex, campus, field of study, occupation, and medical conditions, and convergent validity with the WHOQOL-BREF.

#######################################################################

# Manuscript: Psychometric validation and measurement invariance of the HPLP-II

# in Honduran university students

# Objectives:

# (1) Structural validity & reliability of HPLP-II

# (2) Measurement invariance (sex, campus, field of study, occupation, medical conditions)

# (3) Convergent validity with WHOQOL-BREF

################ 0) PACKAGES ############################################

#######################################################################

# install.packages(c("readxl","psych","lavaan","semPlot","semTools","qgraph","plyr","dplyr",

# "forcats","car","rlang","openxlsx"))

library(readxl)

library(psych)

library(lavaan)

library(semPlot)

library(semTools)

library(openxlsx)

################### 1) DATA ##############################################

# Replace with your local path or use file.choose()

# HPLP_study <- read_excel(file.choose())

HPLP_study <- read_excel("HPLP_II_y_WHOQOL.xlsx")

#######################################################################

################################ 2) CFA: HPLP-II (52 ITEMS)

#######################################################################

# 2.1 Six correlated factors (52 items)

# AF = Physical Activity; CE = Health Care/Stress Management; ME = Emotional Management;

# N = Nutrition; RI = Interpersonal Relations; RS = Health Responsibility

HPLP_6f_52 <- '

AF =~ EVS4 + EVS10 + EVS16 + EVS22 + EVS28 + EVS34 + EVS40 + EVS46

CE =~ EVS6 + EVS12 + EVS18 + EVS24 + EVS30 + EVS36 + EVS42 + EVS48 + EVS52

ME =~ EVS5 + EVS11 + EVS17 + EVS23 + EVS29 + EVS35 + EVS41 + EVS47

N =~ EVS2 + EVS8 + EVS14 + EVS20 + EVS26 + EVS32 + EVS38 + EVS44 + EVS50

RI =~ EVS1 + EVS7 + EVS13 + EVS19 + EVS25 + EVS31 + EVS37 + EVS43 + EVS49

RS =~ EVS3 + EVS9 + EVS15 + EVS21 + EVS27 + EVS33 + EVS39 + EVS45 + EVS51

'

fitHPLP_6f_52 <- sem(HPLP_6f_52, data = HPLP_study, ordered = TRUE, estimator = "WLSMV")

fitMeasures(fitHPLP_6f_52, c("chisq","df","cfi","tli","rmsea","rmsea.ci.lower","rmsea.ci.upper","srmr"))

summary(fitHPLP_6f_52, fit.measures = TRUE, standardized = TRUE)

reliability(fitHPLP_6f_52)

# 2.1.1 Composite reliability #######################

composite_reliability <- function(x, reduce=TRUE, cutoff=0.3){

stopifnot(is.vector(x))

cargas <- abs(x)

if (isTRUE(reduce)) cargas <- cargas[cargas > cutoff]

e <- 1 - cargas^2

(sum(cargas)^2) / ((sum(cargas)^2) + sum(e))

}

cargas_HPLP_6f_52 <- inspect(fitHPLP_6f_52, "std")$lambda

apply(cargas_HPLP_6f_52, 2, composite_reliability)

# 2.2 Second-order model (G defined by AF, CE, ME, N, RI, RS)

HPLP_6f_52_S <- '

AF =~ EVS4 + EVS10 + EVS16 + EVS22 + EVS28 + EVS34 + EVS40 + EVS46

CE =~ EVS6 + EVS12 + EVS18 + EVS24 + EVS30 + EVS36 + EVS42 + EVS48 + EVS52

ME =~ EVS5 + EVS11 + EVS17 + EVS23 + EVS29 + EVS35 + EVS41 + EVS47

N =~ EVS2 + EVS8 + EVS14 + EVS20 + EVS26 + EVS32 + EVS38 + EVS44 + EVS50

RI =~ EVS1 + EVS7 + EVS13 + EVS19 + EVS25 + EVS31 + EVS37 + EVS43 + EVS49

RS =~ EVS3 + EVS9 + EVS15 + EVS21 + EVS27 + EVS33 + EVS39 + EVS45 + EVS51

G =~ AF + CE + ME + N + RI + RS

'

fitHPLP_6f_52_S <- sem(HPLP_6f_52_S, data = HPLP_study, ordered = TRUE, estimator = "WLSMV")

fitMeasures(fitHPLP_6f_52_S, c("chisq","df","cfi","tli","rmsea","rmsea.ci.lower","rmsea.ci.upper","srmr"))

summary(fitHPLP_6f_52_S, fit.measures = TRUE, standardized = TRUE)

reliability(fitHPLP_6f_52_S)

reliabilityL2(fitHPLP_6f_52_S, "G")

# 2.3 Bifactor model (G loads on all items + 6 specific factors)

HPLP_6f_52_bi <- '

AF =~ EVS4 + EVS10 + EVS16 + EVS22 + EVS28 + EVS34 + EVS40 + EVS46

CE =~ EVS6 + EVS12 + EVS18 + EVS24 + EVS30 + EVS36 + EVS42 + EVS48 + EVS52

ME =~ EVS5 + EVS11 + EVS17 + EVS23 + EVS29 + EVS35 + EVS41 + EVS47

N =~ EVS2 + EVS8 + EVS14 + EVS20 + EVS26 + EVS32 + EVS38 + EVS44 + EVS50

RI =~ EVS1 + EVS7 + EVS13 + EVS19 + EVS25 + EVS31 + EVS37 + EVS43 + EVS49

RS =~ EVS3 + EVS9 + EVS15 + EVS21 + EVS27 + EVS33 + EVS39 + EVS45 + EVS51

G =~ EVS1 + EVS2 + EVS3 + EVS4 + EVS5 + EVS6 + EVS7 + EVS8 + EVS9 +

EVS10 + EVS11 + EVS12 + EVS13 + EVS14 + EVS15 + EVS16 + EVS17 + EVS18 +

EVS19 + EVS20 + EVS21 + EVS22 + EVS23 + EVS24 + EVS25 + EVS26 + EVS27 +

EVS28 + EVS29 + EVS30 + EVS31 + EVS32 + EVS33 + EVS34 + EVS35 + EVS36 +

EVS37 + EVS38 + EVS39 + EVS40 + EVS41 + EVS42 + EVS43 + EVS44 + EVS45 +

EVS46 + EVS47 + EVS48 + EVS49 + EVS50 + EVS51 + EVS52

'

fitHPLP_6f_52_bi <- sem(HPLP_6f_52_bi, data = HPLP_study, ordered = TRUE, estimator = "WLSMV")

fitMeasures(fitHPLP_6f_52_bi, c("chisq","df","cfi","tli","rmsea","rmsea.ci.lower","rmsea.ci.upper"))

summary(fitHPLP_6f_52_bi, fit.measures = TRUE, standardized = TRUE)

reliability(fitHPLP_6f_52_bi)

reliabilityL2(fitHPLP_6f_52_bi, "G")

# 2.4 Unidimensional model (52 items)

HPLP_6f_52_uni <- '

G =~ EVS1 + EVS2 + EVS3 + EVS4 + EVS5 + EVS6 + EVS7 + EVS8 + EVS9 +

EVS10 + EVS11 + EVS12 + EVS13 + EVS14 + EVS15 + EVS16 + EVS17 + EVS18 +

EVS19 + EVS20 + EVS21 + EVS22 + EVS23 + EVS24 + EVS25 + EVS26 + EVS27 +

EVS28 + EVS29 + EVS30 + EVS31 + EVS32 + EVS33 + EVS34 + EVS35 + EVS36 +

EVS37 + EVS38 + EVS39 + EVS40 + EVS41 + EVS42 + EVS43 + EVS44 + EVS45 +

EVS46 + EVS47 + EVS48 + EVS49 + EVS50 + EVS51 + EVS52

'

fitHPLP_6f_52_uni <- sem(HPLP_6f_52_uni, data = HPLP_study, ordered = TRUE, estimator = "WLSMV")

fitMeasures(fitHPLP_6f_52_uni, c("chisq","df","cfi","tli","rmsea","rmsea.ci.lower","rmsea.ci.upper","srmr"))

summary(fitHPLP_6f_52_uni, fit.measures = TRUE, standardized = TRUE)

reliability(fitHPLP_6f_52_uni)

reliabilityL2(fitHPLP_6f_52_uni, "G")

#######################################################################

################## 3) MEASUREMENT INVARIANCE ###########################

# Levels: Configural (no constraints) → Metric (loadings) → Scalar (loadings + thresholds)

# Estimation: WLSMV | Ordered items | Base model: HPLP_6f_52

##### 3.1 SEX #####

table(HPLP_study$Sexo_Dico, useNA = "always")

Configural_HPLP_6f_52_sex <- cfa(HPLP_6f_52, data = HPLP_study,

group = "Sexo_Dico",

ordered = TRUE, estimator = "WLSMV")

fitMeasures(Configural_HPLP_6f_52_sex, c("chisq","df","cfi","tli","rmsea","rmsea.ci.lower","rmsea.ci.upper"))

Metric_HPLP_6f_52_sex <- cfa(HPLP_6f_52, data = HPLP_study,

group = "Sexo_Dico",

group.equal = "loadings",

ordered = TRUE, estimator = "WLSMV")

fitMeasures(Metric_HPLP_6f_52_sex, c("chisq","df","cfi","tli","rmsea","rmsea.ci.lower","rmsea.ci.upper"))

Scalar_HPLP_6f_52_sex <- cfa(HPLP_6f_52, data = HPLP_study,

group = "Sexo_Dico",

group.equal = c("loadings","thresholds"),

ordered = TRUE, estimator = "WLSMV")

fitMeasures(Scalar_HPLP_6f_52_sex, c("chisq","df","cfi","tli","rmsea","rmsea.ci.lower","rmsea.ci.upper"))

##### 3.2 UNIVERSITY CAMPUS #####

table(HPLP_study$Campus_universitario, useNA = "always")

Configural_HPLP_6f_52_campus <- cfa(HPLP_6f_52, data = HPLP_study,

group = "Campus_universitario",

ordered = TRUE, estimator = "WLSMV")

fitMeasures(Configural_HPLP_6f_52_campus, c("chisq","df","cfi","tli","rmsea","rmsea.ci.lower","rmsea.ci.upper"))

Metric_HPLP_6f_52_campus <- cfa(HPLP_6f_52, data = HPLP_study,

group = "Campus_universitario",

group.equal = "loadings",

ordered = TRUE, estimator = "WLSMV")

fitMeasures(Metric_HPLP_6f_52_campus, c("chisq","df","cfi","tli","rmsea","rmsea.ci.lower","rmsea.ci.upper"))

Scalar_HPLP_6f_52_campus <- cfa(HPLP_6f_52, data = HPLP_study,

group = "Campus_universitario",

group.equal = c("loadings","thresholds"),

ordered = TRUE, estimator = "WLSMV")

fitMeasures(Scalar_HPLP_6f_52_campus, c("chisq","df","cfi","tli","rmsea","rmsea.ci.lower","rmsea.ci.upper"))

##### 3.3 FIELD OF STUDY #####

table(HPLP_study$AreaConocimiento, useNA = "always")

Configural_HPLP_6f_52_area <- cfa(HPLP_6f_52, data = HPLP_study,

group = "AreaConocimiento",

ordered = TRUE, estimator = "WLSMV")

fitMeasures(Configural_HPLP_6f_52_area, c("chisq","df","cfi","tli","rmsea","rmsea.ci.lower","rmsea.ci.upper"))

Metric_HPLP_6f_52_area <- cfa(HPLP_6f_52, data = HPLP_study,

group = "AreaConocimiento",

group.equal = "loadings",

ordered = TRUE, estimator = "WLSMV")

fitMeasures(Metric_HPLP_6f_52_area, c("chisq","df","cfi","tli","rmsea","rmsea.ci.lower","rmsea.ci.upper"))

Scalar_HPLP_6f_52_area <- cfa(HPLP_6f_52, data = HPLP_study,

group = "AreaConocimiento",

group.equal = c("loadings","thresholds"),

ordered = TRUE, estimator = "WLSMV")

fitMeasures(Scalar_HPLP_6f_52_area, c("chisq","df","cfi","tli","rmsea","rmsea.ci.lower","rmsea.ci.upper"))

##### 3.4 CURRENT OCCUPATION #####

table(HPLP_study$Ocupacion_cat, useNA = "always")

Configural_HPLP_6f_52_ocup <- cfa(HPLP_6f_52, data = HPLP_study,

group = "Ocupacion_cat",

ordered = TRUE, estimator = "WLSMV")

fitMeasures(Configural_HPLP_6f_52_ocup, c("chisq","df","cfi","tli","rmsea","rmsea.ci.lower","rmsea.ci.upper"))

Metric_HPLP_6f_52_ocup <- cfa(HPLP_6f_52, data = HPLP_study,

group = "Ocupacion_cat",

group.equal = "loadings",

ordered = TRUE, estimator = "WLSMV")

fitMeasures(Metric_HPLP_6f_52_ocup, c("chisq","df","cfi","tli","rmsea","rmsea.ci.lower","rmsea.ci.upper"))

Scalar_HPLP_6f_52_ocup <- cfa(HPLP_6f_52, data = HPLP_study,

group = "Ocupacion_cat",

group.equal = c("loadings","thresholds"),

ordered = TRUE, estimator = "WLSMV")

fitMeasures(Scalar_HPLP_6f_52_ocup, c("chisq","df","cfi","tli","rmsea","rmsea.ci.lower","rmsea.ci.upper"))

##### 3.5 SELF-REPORTED PREEXISTING MEDICAL CONDITIONS #####

table(HPLP_study$Enfermedades_prexistentes2, useNA = "always")

Configural_HPLP_6f_52_enf <- cfa(HPLP_6f_52, data = HPLP_study,

group = "Enfermedades_prexistentes2",

ordered = TRUE, estimator = "WLSMV")

fitMeasures(Configural_HPLP_6f_52_enf, c("chisq","df","cfi","tli","rmsea","rmsea.ci.lower","rmsea.ci.upper"))

Metric_HPLP_6f_52_enf <- cfa(HPLP_6f_52, data = HPLP_study,

group = "Enfermedades_prexistentes2",

group.equal = "loadings",

ordered = TRUE, estimator = "WLSMV")

fitMeasures(Metric_HPLP_6f_52_enf, c("chisq","df","cfi","tli","rmsea","rmsea.ci.lower","rmsea.ci.upper"))

Scalar_HPLP_6f_52_enf <- cfa(HPLP_6f_52, data = HPLP_study,

group = "Enfermedades_prexistentes2",

group.equal = c("loadings","thresholds"),

ordered = TRUE, estimator = "WLSMV")

fitMeasures(Scalar_HPLP_6f_52_enf, c("chisq","df","cfi","tli","rmsea","rmsea.ci.lower","rmsea.ci.upper"))

##################################################################################### 4) WHOQOL-BREF: MEASUREMENT MODEL #####################

WHOQOL_4f <- '

F =~ CV_3i + CV_4i + CV_10 + CV_15 + CV_16 + CV_17 + CV_18

Ps =~ CV_5 + CV_6 + CV_7 + CV_11 + CV_19 + CV_26i

RS =~ CV_20 + CV_21 + CV_22

Amb =~ CV_8 + CV_9 + CV_12 + CV_13 + CV_14 + CV_23 + CV_24 + CV_25

'

fitWHOQOL_4f <- sem(WHOQOL_4f, data = HPLP_study, ordered = TRUE, estimator = "WLSMV")

fitMeasures(fitWHOQOL_4f, c("chisq","df","cfi","tli","rmsea","rmsea.ci.lower","rmsea.ci.upper","srmr"))

summary(fitWHOQOL_4f, fit.measures = TRUE, standardized = TRUE)

reliability(fitWHOQOL_4f)

############################################################################### 5) CONVERGENT VALIDITY: CORRELATIONS HPLP-II ↔ WHOQOL-BREF ######

#######################################################################

modelo_HPLP52_WHOQOL <- '

# WHOQOL factors

F =~ CV_3i + CV_4i + CV_10 + CV_15 + CV_16 + CV_17 + CV_18

Ps =~ CV_5 + CV_6 + CV_7 + CV_11 + CV_19 + CV_26i

RS =~ CV_20 + CV_21 + CV_22

Amb =~ CV_8 + CV_9 + CV_12 + CV_13 + CV_14 + CV_23 + CV_24 + CV_25

# HPLP-II factors (52-item, 6 correlated factors)

AF =~ EVS4 + EVS10 + EVS16 + EVS22 + EVS28 + EVS34 + EVS40 + EVS46

CE =~ EVS6 + EVS12 + EVS18 + EVS24 + EVS30 + EVS36 + EVS42 + EVS48 + EVS52

ME =~ EVS5 + EVS11 + EVS17 + EVS23 + EVS29 + EVS35 + EVS41 + EVS47

N =~ EVS2 + EVS8 + EVS14 + EVS20 + EVS26 + EVS32 + EVS38 + EVS44 + EVS50

RI =~ EVS1 + EVS7 + EVS13 + EVS19 + EVS25 + EVS31 + EVS37 + EVS43 + EVS49

RS =~ EVS3 + EVS9 + EVS15 + EVS21 + EVS27 + EVS33 + EVS39 + EVS45 + EVS51

# Cross-block correlations (evidence of convergence)

F ~~ AF + CE + ME + N + RI + RS

Ps ~~ AF + CE + ME + N + RI + RS

RS ~~ AF + CE + ME + N + RI + RS

Amb~~ AF + CE + ME + N + RI + RS

'

fitmodelo_HPLP52_WHOQOL <- sem(modelo_HPLP52_WHOQOL, data = HPLP_study, ordered = TRUE, estimator = "WLSMV")

fitMeasures(fitmodelo_HPLP52_WHOQOL, c("chisq","df","cfi","tli","rmsea","rmsea.ci.lower","rmsea.ci.upper","srmr"))

summary(fitmodelo_HPLP52_WHOQOL, fit.measures = TRUE, standardized = TRUE)

##############################################################################################################################################
